# Migrasomes from adipose derived stem cells enrich CXCL12 to recruit stem cells via CXCR4/RhoA for a positive feedback loop mediating soft tissue regeneration

**DOI:** 10.1186/s12951-024-02482-9

**Published:** 2024-05-03

**Authors:** Yunzi Chen, Ye Li, Bin Li, Delin Hu, Ziqing Dong, Feng Lu

**Affiliations:** Department of Plastic and Cosmetic Surgery, Nanfang Hospital, Southern Medical University, 1838 Guangzhou North Road, Guangzhou, Guangdong 510515 P.R. China

**Keywords:** Adipose derived stem cells, Migrasomes, Stem cell recruitment, CXCL12, CXCR4/RhoA, Soft tissue regeneration

## Abstract

**Background:**

Adipose-derived stem cells (ASCs) represent the most advantageous choice for soft tissue regeneration. Studies proved the recruitment of ASCs post tissue injury was mediated by chemokine CXCL12, but the mechanism by which CXCL12 is generated after tissue injury remains unclear. Migrasomes are newly discovered membrane-bound organelles that could deliver CXCL12 spatially and temporally in vivo. In this study, we sought to investigate whether migrasomes participate ASC-mediated tissue regeneration.

**Methods:**

Discrepant and asymmetrical soft tissue regeneration mice model were established, in which HE staining, immunofluorescent staining, western blot and qPCR were conducted to confirm the role of CXCL12 and migrasomes in ASC-mediated tissue regeneration. Characterization of ASC-derived migrasomes were carried out by confocal microscopy, scanning electron microscopy, transmission electron microscopy as well as western blot analysis. The function and mechanism of migrasomes were further testified by assisting tissue regeneration with isolated migrasomes in vivo and by in vitro transwell combined with co-culture system.

**Results:**

Here, we show for the first time that migrasomes participate in soft tissue regeneration. ASCs generate migrasomes enriched with CXCL12 to mediate tissue regeneration. Migrasomes from ASCs could promote stem cells migration by activating CXCR4/RhoA signaling in vivo and in vitro. Chemoattracted ASCs facilitate regeneration, as demonstrated by the upregulation of an adipogenesis-associated protein. This positive feed-back-loop creates a favorable microenvironment for soft tissue regeneration. Thus, migrasomes represent a new therapeutic target for ASC-mediated tissue regeneration.

**Conclusions:**

Our findings reveal a previously unknown function of ASCs in mediating tissue regeneration by generating migrasomes. The ASC-derived migrasomes can restore tissue regeneration by recruiting stem cells, which highlighting the potential application of ASC-derived migrasomes in regenerative medicine.

**Supplementary Information:**

The online version contains supplementary material available at 10.1186/s12951-024-02482-9.

## Introduction

Soft tissue defects, such as those resulted from traumatic resection of tumor, congenital diseases, bring about sufferings not only cosmetically but also functionally as well as mentally. Adipose tissue represents the most natural filler to restore volume defects with additional benefits of alleviating fibrosis, pain, and skin refinement [1–3]. Augmentation of adipose tissue regeneration in situ and post-transplantation has gained great attention in regenerative medicine [4, 5].

Adipose-derived stem cells (ASCs) are the mesenchymal stem cells found within adipose tissue and are the main contributors to regenerative events of adipose tissue. Native ASCs express CD34 as cell marker in adipose tissue and the number of endogenous CD34^+^ ASCs within adipose tissue strongly correlated with the outcomes of tissue regeneration [6–8]. However, tissue-resident stem cells are generally insufficient to achieve ideal outcomes owing to the harsh environment and limited cell quantities, leading to the development of strategies such as cell-assisted lipotransfer and mobilization of endogenous ASCs [9, 10]. Stem cell-based regeneration requires migration of ASCs to the damaged site. We and others have shown that upregulation of the local chemokine CXCL12 after tissue injury contributes to recruitment of ASCs through the receptor CXCR4 [11, 12]. Attempts to facilitate tissue regeneration targeting CXCL12 to attract ASCs have yield promising results [13, 14]. Thus, elucidation of the mechanism by which CXCL12 is generated after adipose tissue injury may help to identify pathways that mediate migration of ASCs and potential therapeutic strategies for boosting ASC-mediated tissue regeneration.

To establish a chemokine gradient, the prevailing diffusion model might agree with the simplest circumstances only, whereas upon inflammation or tissue damage, chemokines may be degraded and not survive the harsh milieu consisting of scavenger receptors and extracellular enzymes [15, 16]. Packaging signals into membrane vesicles alleviates the need for diffusion-based dispersal of chemokines and has been reported during tissue development [15, 17–19]. Migrasomes are newly discovered membrane-bound organelles generated on retraction fibers of migrating cells [20]. Chemical signals packaged into migrasomes can be delivered in a spatial and temporal-dependent manner. This novel type of signaling mechanism establishes a localized CXCL12 signal to chemoattract CXCR4^+^ monocytes during angiogenesis in chicken embryonic model and or dorsal forerunner cells during organ morphogenesis of zebrafish gastrulation [19, 21]. Recent study also revealed that migrasoems mediated communications between stem cells, as migrasomes containing CXCL12 from bone marrow derived mesenchymal stem cells could chemoattracted CD34^+^ hematopoietic stem cells [22]. Thus, we speculated that CXCL12 is delivered by migrasomes to promote recruitment of ASCs during adipose tissue regeneration.

In this study, we identified migrasomes participate in adipose tissue regeneration for the first time. ASCs generate migrasomes enriched with CXCL12 to promote ASC recruitment in vivo and in vitro. In terms of mechanism, migrasomes mediate stem cell migration through activating CXCR4/RhoA signal. Chemoattracted ASCs facilitate regeneration, as demonstrated by the upregulation of adipogenesis-associated protein. This positive feed-back-loop creates a favorable microenvironment for adipose tissue regeneration. Thus, migrasomes represent a new therapeutic target for ASC-mediated tissue regeneration.

## Materials and methods

### Antibodies and reagents

Anti-TSPAN4 (Biossusa, Cat#bs-9413R); anti-TSPAN7 (Biorbyt, Cat#orb373388); anti-integrin β1 (SantaCruz, Cat#sc374429); anti-CXCL12 (Proteintech, Cat#174021AP); anti-CXCR4 (Abcam, Cat#ab124824); anti-CD34 (Abcam, Cat#ab81289); anti-Perilipin (Progen, Cat#Gp40); anti-RhoA (Abcam, Cat#ab86297); anti-PPARγ (Abcam, Cat# ab209350) antibodies; goat anti-rabbit Alexa Fluor® 488 IgG (Abcam, Cat# ab150077); goat anti-guinea pig Alexa Fluor® 647 IgG (Abcam, Cat# ab150187); goat anti-rabbit Alexa Fluor® 647 IgG (Abcam, Cat#ab150079); goat anti-mouse Alexa Fluor® 488 IgG (Abcam, Cat#ab150113); AMD3100 (Abmole, Cat#M1898); CCG-1423 (Abmole, Cat#M8999); culture medium for mouse ASCs (OriCell, Cat#MUXMD9011); PBS (Gibco, Cat#10,010,023); trypsin–EDTA (Gibco, Cat#25,200,056); siRNA targeting CXCL12 (SANTA CRUZ, Cat#sc-39,368).

### Mice

All animal experiments were approved by the Nanfang Hospital Animal Ethics Committee Laboratory and were conducted according to the guidelines of the National Health and Medical Research Council of China. Male C57/BL6 mice with an average weight of 20 g were obtained from Southern Medical University, housed in individual cages with a 12 h light/dark cycle and fed standard food and water ad libitum.

### Mouse fat grafting model with a donor site and Normal/Ischemic adipose tissue regeneration mouse model

Seventy-two C57/BL6 mice were used to build mouse fat grafting model with a donor site. To prepare grafts, 30 mice were anesthetized by intraperitoneal injection of pentobarbital sodium (50 mg/kg, Nembutal) and the bilateral subcutaneous inguinal fat pads were separated and removed. The removed adipose tissue was placed in culture dishes on ice and gently cut into very small pieces similar to the size of clinically used aspirated fat tissue with corneal scissors. A volume of 0.3mL prepared adipose tissue served as the baseline fat grafts for each mouse and was injected within 30 min post-harvesting.

**Fig. 1:**
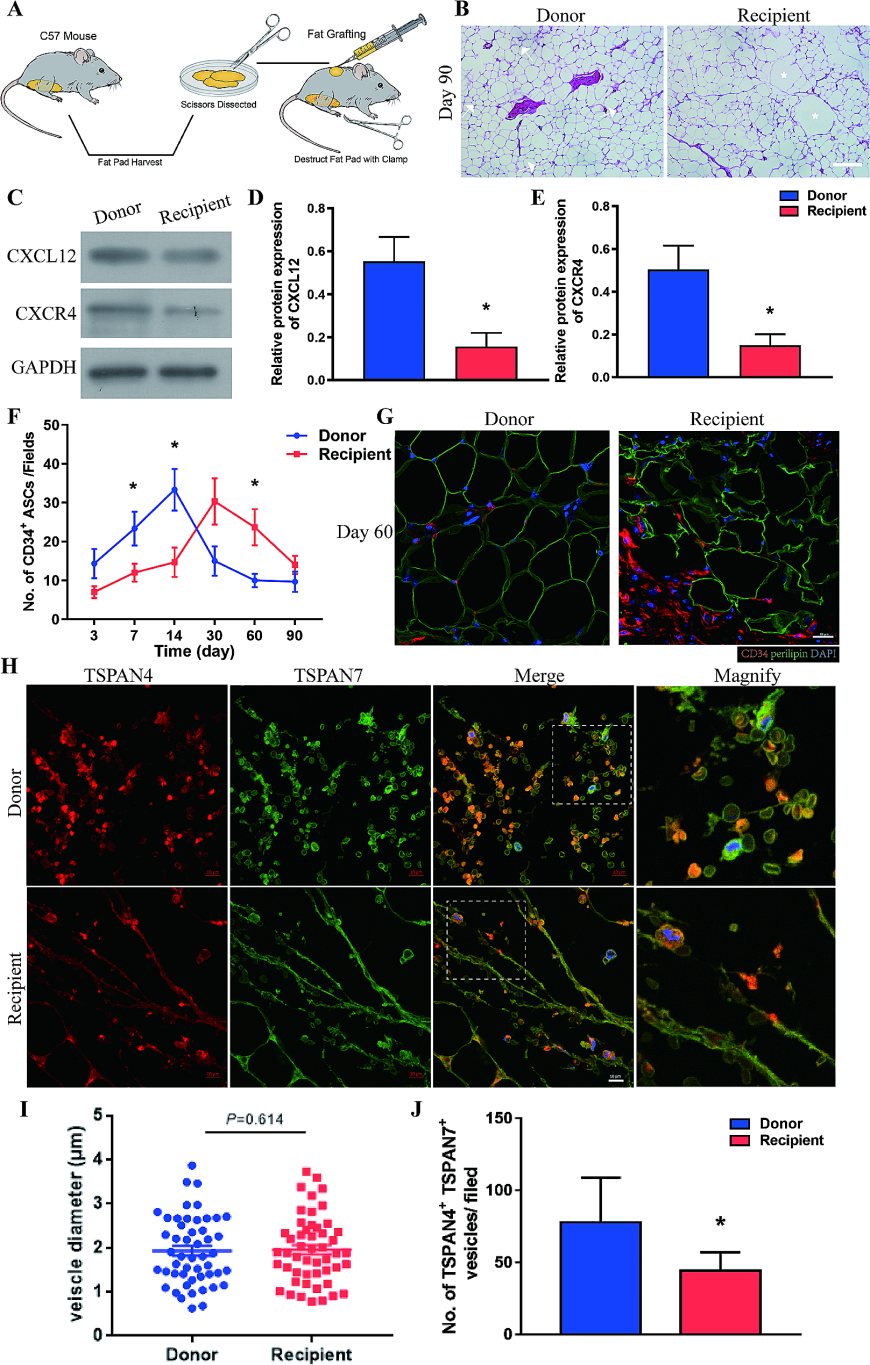
Recruitment of ASCs by CXCL12 mediates adipose tissue regeneration. (**A**) Schematic illustration of the novel mouse fat grafting model with a donor site. *N* = 7 in each groups. (**B**) HE staining of adipose tissue obtained from the donor and recipient sites 90 days after surgery. Scale bar, 50 μm. White arrows indicate small adipocytes and asterisks indicate vacuoles. (**C**) Western blot analysis of CXCL12 and CXCR4 protein expression in adipose tissue from the donor and recipient sites 3 days after surgery. (**D-E**) Semi-quantitative analysis of CXCL12 and CXCR4 protein expression. *p < 0.05 compared with Donor. (**F**) Quantitative analysis of infiltrated CD34^+^ ASCs per field at the donor and recipient site over time. *p < 0.05 compared with Donor. (**G**) Immunofluorescence staining of CD34 and perilipin in adipose tissue from the donor and recipient site at 60 days after surgery. Scale bar, 20 μm. (**H**) Immunofluorescence stanning of TSPAN4 and TSPAN7 in adipose tissue from donor and recipient sites at 3 days after surgery. Scale bar, 10 μm. (**I**) Diameters of the TSPAN4^+^ and TSPAN7^+^ vesicles. *n* = 50 vesicles per group. (**J**) Quantitative analysis of TSPAN4^+^ and TSPAN7^+^ vesicles per field in adipose tissue from the donor and recipient site at 3 days after surgery. *p < 0.05 compared with Donor. The data mean ± SEM. Statistical differences in (D), (E), (I) and (J) were assessed with Student’s t test, (F) was analyzed with One-way ANOVA

For the mouse fat grafting model with a donor site, 42 mice were anesthetized, and the inguinal skin was incised. To establish the donor site, the subcutaneous inguinal fat pads were dissected and crushed using a clamp as previously described [23, 24], and the skin was closed with 7 − 0 nylon sutures. Each mouse was subcutaneously injected with 0.3mL prepared fat graft on the back (recipient site) using a 1mL syringe and an 18-gauge cannula (Fig. 1A). Seven animals were sacrificed at 3, 7, 14, 30, 60, and 90 days after surgery.

**Fig. 2:**
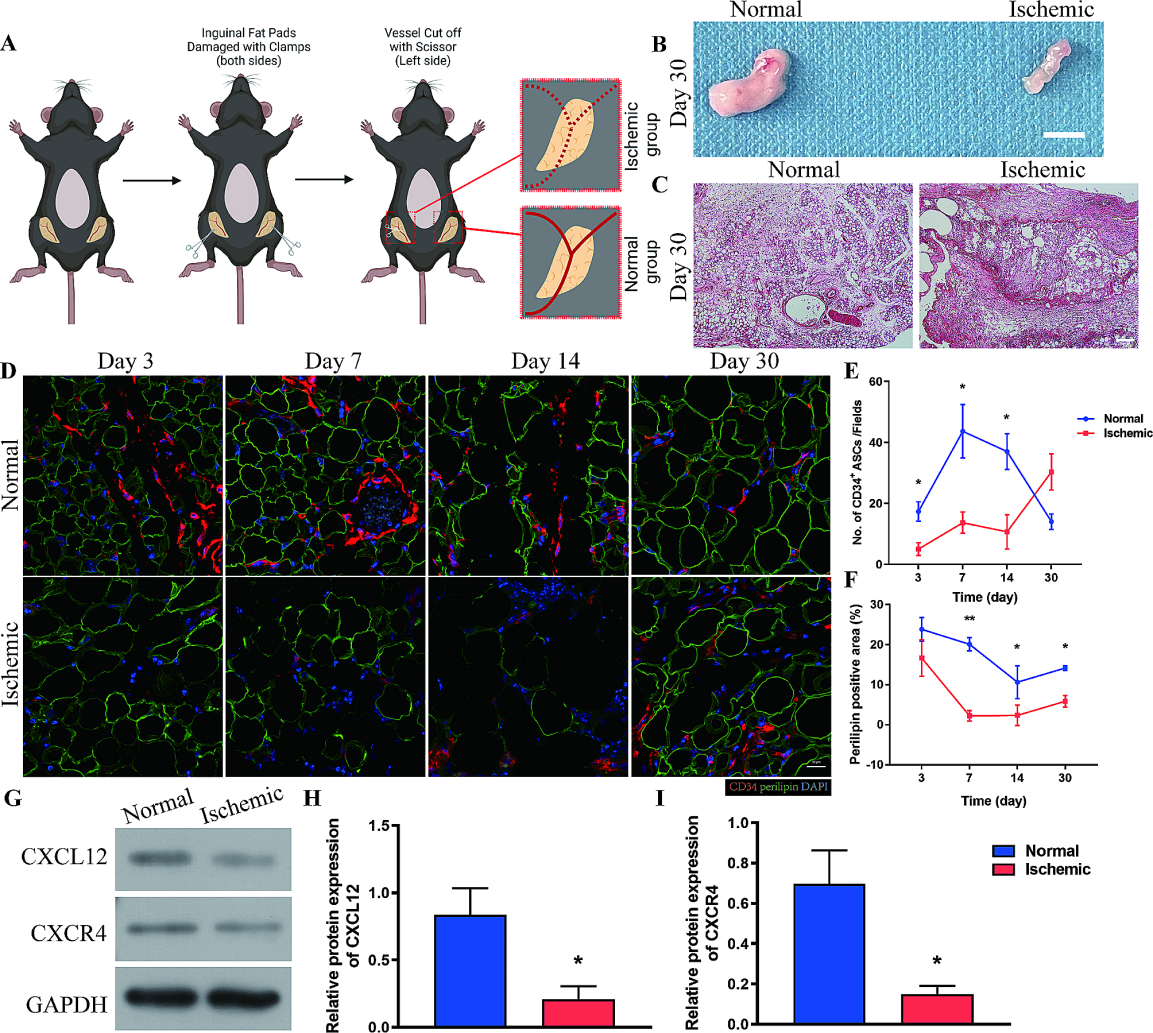
CXCL12 mediates asymmetric adipose tissue regeneration. (**A**) Schematic illustration of the mouse asymmetric adipose tissue regeneration model. *N* = 7. (**B**) Macroscopic observation of adipose tissue from the Normal and Ischemic groups at 30 days after surgery. Scale bar, 50 mm. (**C**) HE staining of adipose tissue from the Normal and Ischemic groups at 30 days after surgery. Scale bar, 200 μm. (**D**) Immunofluorescence staining of CD34 and perilipin in adipose tissue from Normal and Ischemic groups over time. Scale bar, 20 μm. (**E**) Quantitative analysis of infiltrated CD34^+^ ASCs per field in adipose tissue over time. *p < 0.05 compared with Normal. (**F**) Quantitative analysis of perilipin^+^ area per field over time. *p < 0.05, **p < 0.01 compared with Normal. (**G**) Western blot analysis of CXCL12 and CXCR4 protein expression in adipose tissue from the Normal and Ischemic groups at 7 days after surgery. (**H-I**) Semiquantitative analysis of CXCL12 and CXCR4 protein expression. *p < 0.05 compared with Normal. The data are mean ± SEM. Statistical differences in (E) and (F) were assessed with One-way ANOVA. (H) and (I) were analyzed using Student’s t test

Another 42 mice were used to generate the Normal/Ischemic adipose tissue regeneration model. The bilateral inguinal skin was incised, and the inguinal fat pads were damaged using a clamp. The inguinal vessels were left intact on the right side (Normal site) and cut using scissors on the left side (Ischemic site). After surgery, the skin was closed with 7 − 0 nylon sutures (Fig. 2A). Seven animals were sacrificed at 1, 3, 5, 7, 14 and 30 days after surgery.

Adipose tissue was carefully separated from the surrounding tissue and each sample was assessed by histology, immunofluorescence, western blotting and qPCR.

### Isolation and culture of ASCs

ASCs were isolated from inguinal adipose tissue of 15 healthy C57/BL6 mice. Mice were anesthetized and adipose tissue was obtained from the bilateral inguinal fat pads. The harvested adipose tissue was finely minced and digested with PBS containing collagenase (Solarbio, Cat#C8140) in a 50mL centrifuge tube on a shaker for 40 min at 37℃. The digested tissue was centrifuged at 1500 rpm for 5 min and filtered to remove large debris. The cellular pellet (stromal vascular fraction) was resuspended in erythrocyte lysis buffer (Solarbio, Cat#R1010), and centrifuged at 1000 rpm for 5 min followed by plating in 25cm^2^ flasks. The cells were cultured on plastic culture dishes using culture medium for mouse ASCs supplemented with 1% 100 U penicillin/100 U streptomycin, and 10% serum for mouse ASCs. Cells were used at passage 3–5.

ASCs were harvested with 0.05% Trypsin–EDTA (Gibco), washed 3 times with PBS and prepared as cell suspension in culture medium with 10% serum. Cells were seeded as suspension into 6-well plates at the density of 2.5 × 10^5^ per well (triplicate wells for each condition) and incubate at 37 °C in 5% CO2 for 24 h. For stimulatory experiments, 400µL migrasomes (10 mg/mL) was added to the plates for the next two continuous days. And for inhibition experiments, 400µL AMD3100 (30µM), or 400µL CCG-1423 (300nM, Abmole) were added for two consecutive days. Cells were incubated for another 24 h and samples were collected at the fourth day for western blot and qPCR analysis.

### siRNA knockdown of CXCL12

ASCs were seeded as suspension into 6-well plates and incubate at 37 °C in 5% CO2 for 24 h. Cells were transfected with a non-targeting siRNA or siRNAs targeting CXCL12 according to the manufacturer’s instruction.

### Isolation and characterization of migrasomes

ASCs were cultured on culture dishes. Migrasomes produced by ASCs were isolated by sequential centrifugation as previously described [21, 25]. Cells were harvested with 0.05% trypsin–EDTA after removal of the supernatant, collected in 50mL tubes, and centrifuged at 1000 g for 10 min to remove cell bodies and then at 4000 g for 20 min to remove cell fragments. Crude migrasomes were collected as the pellet by centrifuged at 20,000 g for 30 min. Isolated migrasomes were subjected to biological and morphological analyses.

### Electron microscopy

For scanning electron microscopy, ASCs were cultured on coverslips, and fixed with 2.5% glutaraldehyde in PBS for 2 h at room temperature. Fixed cells were washed three times with PBS and post-fixed with 1% osmium containing 1.5% potassium ferrocyanide for 60 min at room temperature. All samples were then dehydrated with a graded ethanol series (50%, 70%, 90%, 95%, and 100%) for 8 min each. After changing ethanol for tertiary butyl alcohol, samples were frozen at − 20 °C and dried with a freeze drier. Dried samples were coated with an ∼ 10-nm thick gold film by sputter coating before examination with a field emission scanning electron microscope.

For Transmission electron microscopy (TEM), cells were cultured on 6-well plates for 24 h, scratched using cell scraper, and centrifuged at 1500 rpm for 20 min to form a cell cluster. The cell cluster was fixed with 2.5% glutaraldehyde in PBS for 2 h at room temperature, followed by dehydration with a graded ethanol series (50%, 70%, 90%, 95%, and 100%) for 8 min each. Samples were infiltrated with and embedded in SPON12 resin. After polymerization for 48 h at 60 °C, ultrathin (70 nm thick) sections were cut using a diamond knife, picked up with Formvar-coated copper grids (100 mesh), and double stained with uranyl acetate and lead citrate. After air drying, samples were examined with a transmission electron microscope.

### Delivery of migrasomes in the ischemic adipose tissue regeneration mouse model

Ischemic adipose tissue regeneration mouse model was generated as described above. Briefly, the bilateral inguinal fat pads were damaged using a clamp and the inguinal vessels on both sides were cut. Thereafter, 30µL of migrasome sediment (100 µg) was injected into the left site (+ Mig group) and an equal volume of PBS was injected into the right site (PBS group). Seven animals were sacrificed at 1, 3, 5, 7, 14, and 30 days after surgery. Adipose tissue at both sites was carefully separated from the surrounding tissue. Each sample was assessed by histology, immunofluorescence, western blotting and qPCR.

### Inhibition of ASCs infiltration in normal regeneration model

Normal adipose tissue regeneration mouse model was generated as described above. Briefly, the bilateral inguinal fat pads were damaged using a clamp and the inguinal vessels on both sides were left intact. Thereafter, 30µL of AMD3100 (5 mg/kg) was injected into the left site (+ AMD group) to inhibit infiltration of ASCs and an equal volume of PBS was injected into the right site (PBS group). Seven animals were sacrificed at 1, 3, 5, 7, 14, and 30 days after surgery. Adipose tissue at both sites was carefully separated from the surrounding tissue. Each sample was assessed by histology, immunofluorescence, western blotting and qPCR.

### Histological examination

Tissue samples were fixed in 4% paraformaldehyde overnight, dehydrated using a graded alcohol series, then embedded in paraffin, and processed to obtain tissue sections for HE staining. Images were obtained using an Axio Image D2 microscope (Zeiss) and photographed using Axio Image D2 digital camera.

### Immunofluorescence staining

Tissue sections were deparaffinized, rehydrated, and washed three times with Tris-buffered saline (Sigma). Sections were incubated with H_2_O_2_ for 30 min and washed with distilled water to avoid nonspecific background staining. Adipose tissue samples were stained with anti-CD34 (Abcam) and guinea pig anti‐mouse perilipin (Progen) antibodies following the manufacturers’ instructions. After washing, the samples were incubated with goat anti-rabbit Alexa Fluor® 488 IgG (Abcam) and goat anti-guinea pig Alexa Fluor® 647 IgG (Abcam). Nuclei were stained with DAPI (Sigma, USA).

For migrasomes detection, tissue samples were stained with anti-TSPAN4 (Biossusa), anti-TSPAN7 (Biorbyt) antibodies following the manufacturers’ instructions and then with goat anti-rabbit Alexa Fluor® 488 IgG (Abcam) and goat anti-rabbit Alexa Fluor® 647 IgG (Abcam). Nuclei were stained with DAPI (Sigma, USA). Furthermore, the tissue samples were stained with anti-integrin β1 (SantaCruz), and then with goat anti-mouse Alexa Fluor® 488 IgG (Abcam). Nuclei were stained with DAPI (Sigma). Images were obtained using Axio Image D2 microscope (Zeiss) and analyzed using ImageJ software.

### Transwell assay

Transwell inserts (8 μm plate, Costar 3422) were pre-incubated with serum-free culture medium at 37 °C. ASCs were harvested with 0.05% trypsin–EDTA, washed three times with PBS and suspended in serum-free culture medium. About 1 × 10^5^ cells in 200µL were seeded into the upper chamber of each transwell (triplicate wells for each condition). At the same time, 300µL ASC culture medium containing 10% serum was placed in the lower chamber as the control group. For chemotaxis experiments, 200µL migrasomes (10 mg/mL) was added to the lower chamber. For inhibitor experiments, 200µL AMD3100 (30µM), or CCG-1423 (300nM) was added to the lower chamber. For rescue experiments, 20 µg migrasomes and the inhibitor were placed in the lower chamber. The plates were then incubated at 37 °C in 5% CO_2_ for 12 h and 24 h, and cells were fixed relatively with 4% paraformaldehyde and stained with crystal violet. Cells that did not migrate through were gently removed using a cotton swab. Migrated ASCs were visualized using a light microscope and analyzed using ImageJ software.

### Western blot analysis

Total cell lysates were prepared using M-PER Mammalian Protein Extraction Reagent (ThermoFisher Scientific, Cat#78,503). A bicinchoninic acid protein assay (ThermoFisher Scientific) was used to estimate protein concentration. After separation by sodium dodecyl sulfate-polyacrylamide gel electrophoresis using a NuPAGE electrophoresis system, protein extracts were transferred to immobilon polyvinylidene difluoride membranes. The membranes were blocked in 5% milk and then incubated with anti-CXCL12 (Proteintech), anti-CXCR4 (Abcam), anti-TSPAN4 (Biossusa), anti-integrin β1 (Abcam), anti-actin (Abcam), anti-RhoA, and anti-PPAR-γ (Abcam) primary antibodies following the manufacturers’ instructions. Thereafter, the membranes were incubated with secondary antibodies and a WesternBreeze Chemiluminescent Detection Kit (ThermoFisher Scientific) was used to detect signals. Glyceraldehyde-3-phosphate dehydrogenase (GAPDH) served as an internal control.

### Quantitative reverse transcription PCR

Total RNA was isolated from tissue samples using RNAiso (TaKaRa) and from ASCs using TRIzol-Reagent (Invitrogen). The concentration of total RNA was determined using BioPhotometer plus (Eppendorf). cDNA was synthesized using EasyScript First-Strand cDNA Synthesis SuperMix and amplified over 40 cycles using SYBR Green qPCR SuperMix (Vazyme) and an ABI PRISM® 7500 Sequence Detection System. All experiments were performed in triplicate to obtain average data. The primer sequences are shown in Table Supplementary 1.

### Statistical analysis

Statistical analysis was performed using the Statistical Package for the Social Sciences (IBM SPSS ver. 21.0; IBM Corp., Armonk, NY, USA). Test of normality was performed before the analysis of Student’s t test and one-way ANOVA followed by post-test. *p* < 0.05 was considered significant.

## Results

### CXCL12 mediates adipose tissue regeneration by recruiting ASCs via migrasomes

To elucidate the mechanism underlying soft tissue regeneration, we utilized our previously established fat grafting model with a donor site [23, 24] (Fig. 1A). This model optimizes the exploration on adipose regeneration by better mimicking the situation of clinical autologous fat grafting which contains fat harvesting (donor site) and fat grafting (recipient site). Adipose tissues at the donor and recipient sites in this model showed different repair and regeneration outcomes, with tissues of the donor site repaired better than that at the recipient site. And the mechanism behind this discrepant regeneration process has been proved to be ascribed to the different expression of CXCL12 post tissue damage [24].

Herein, in same mice model, hematoxylin and eosin (HE) analysis at 90 days after grafting showed that mature adipocytes were regularly arranged, small adipocytes (white arrows) were scattered throughout, and the tissue structure was stable at the donor site, while broken cells, vacuoles (asterisk), and severe tissue fibrosis were observed at the recipient site (Fig. 1B). Western blot analysis showed that protein expression of CXCL12 and CXCR4 was higher at the donor site than at the recipient site as early as Day3 after tissue injury (Fig. 1C-E). Quantitative analysis of immunofluorescence staining of CD34 showed that the number of CD34^+^ ASCs at the donor site was higher than that at the recipient site soon after injury, increased from Day3 to Day 14, and decreased thereafter. However, infiltration of ASCs was delayed at the recipient site. The number of CD34^+^ ASCs at the recipient site was lower than that at the donor site during the first 14 days, increased from Day 14 to Day 30, and decreased thereafter (Fig. 1F). Significantly more CD34^+^ ASCs infiltrated the recipient site than the donor site at the later stage of tissue repair, indicating that regeneration was incomplete. By contrast, there were fewer ASCs at the donor site and perilipin staining showed that the tissue structure was integrated (Fig. 1G).

To investigate whether migrasomes participate in tissue repair, expression of the migrasome markers TSPAN4 and TSPAN7 was tested in tissue samples from Day 3. Immunofluorescence staining showed that vesicles enriched with TSPAN4 and TSPAN7 were present at both the donor and recipient sites. Magnified images showed that some of these vesicles were attached to tubular structures that extended from cells, while others were scattered among cells (Fig. 1H). The diameters of these vesicles were mostly 0.5–3 μm, which is consistent with the characterizations of migrasomes [19–21] (Fig. 1I). Furthermore, there were significantly more migrasome-like vesicles at the donor site than at the recipient site soon after fat grafting (Fig. 1J).

These findings showed that the chemokine CXCL12 might mediate infiltration of ASCs during adipose tissue regeneration and that migrasomes might be involved in expression of CXCL12.

Figure 1. **Recruitment of ASCs by CXCL12 mediates adipose tissue regeneration. (A)** Schematic illustration of the novel mouse fat grafting model with a donor site. *N* = 7 in each groups. (**B**) HE staining of adipose tissue obtained from the donor and recipient sites 90 days after surgery. Scale bar, 50 μm. White arrows indicate small adipocytes and asterisks indicate vacuoles. (**C**) Western blot analysis of CXCL12 and CXCR4 protein expression in adipose tissue from the donor and recipient sites 3 days after surgery. (**D-E**) Semi-quantitative analysis of CXCL12 and CXCR4 protein expression. *p < 0.05 compared with Donor. (**F**) Quantitative analysis of infiltrated CD34^+^ ASCs per field at the donor and recipient site over time. *p < 0.05 compared with Donor. (**G**) Immunofluorescence staining of CD34 and perilipin in adipose tissue from the donor and recipient site at 60 days after surgery. Scale bar, 20 μm. (**H**) Immunofluorescence stanning of TSPAN4 and TSPAN7 in adipose tissue from donor and recipient sites at 3 days after surgery. Scale bar, 10 μm. (**I**) Diameters of the TSPAN4^+^ and TSPAN7^+^ vesicles. *n* = 50 vesicles per group. (**J**) Quantitative analysis of TSPAN4^+^ and TSPAN7^+^ vesicles per field in adipose tissue from the donor and recipient site at 3 days after surgery. *p < 0.05 compared with Donor. The data mean ± SEM. Statistical differences in (D), (E), (I) and (J) were assessed with Student’s t test, (F) was analyzed with One-way ANOVA.

### CXCL12 regulates recruitment of ASCs during asymmetric adipose tissue regeneration

To further look into the mechanism behind the different expression of CXCL12 between the donor and recipient site, we sought to diminish the bias brought by physiological environment. Thus, we transferred the adipose regeneration model on the bilateral inguinal fat pads of mice. To further mimic the damage pattern of donor and recipient site, we manipulated the inguinal vessels considering the most prominent difference between the donor and the recipient site of fat grafting is that the vascular honeycomb structure is intact at aspirated sites after liposuction while grafts at recipient sites are avascular [26]. This asymmetric adipose regeneration model was well-established by the published work to evaluate the regeneration of adipose tissue and the activity of ASCs post tissue damage [27, 28]. To this end, the inguinal vessels were left intact on the right side to mimic the donor site (Normal) and cut with scissors on the left side to mimic the recipient site (Ischemic) (Fig. 2A).

As expected, macroscopic observation on Day30 showed that tissues had a natural appearance and soft texture in the Normal group, but appeared atrophic and brittle in the Ischemic group (Fig. 2B). Histological analysis showed that differently sized adipocytes were tightly arranged in the Normal group, whereas large vacuoles and severe fibrosis were observed in the Ischemic group (Fig. 2C). Immunofluorescence staining of CD34 showed that significantly more ASCs infiltrated tissues in the Normal group than in the Ischemic group as early as Day3 post injury, and the number of ASCs in the Normal group kept rising from Day3 to Day7 and decreased thereafter. However, infiltration of ASCs was delayed in the Ischemic group. The number of infiltrated ASCs in the Ischemic group remained relatively low during the first 14 days post-injury and increased sharply from Day 14 to Day 30 (Fig. 2D, E). Perilipin is only detectable in viable adipocytes. Staining of perilipin showed that the adipose tissue structure was relatively stable during the first 7 days post-injury in the Normal group, whereas the perilipin^+^ area sharply decreased in the Ischemic group. From Day 7 to Day 14, the perilipin^+^ area decreased in the Normal group but was stably small in the Ischemic group. From Day 14, tissue regeneration was observed in both groups, as shown by the increased perilipin^+^ area. Nevertheless, Nevertheless, the perilipin^+^ area was significantly larger, and the number of infiltrated ASCs was lower in the Normal group than in the Ischemic group on Day 30, suggesting that regeneration was better in the Normal group (Fig. 2D, F).

To further investigate the mechanism underlying the asymmetric regeneration between the Normal and Ischemic groups, we tested expression of CXCL12 and CXCR4. Quantitative PCR (qPCR) analysis showed that expression of CXCL12 and CXCR4 was higher in the Normal group than in the Ischemic group soon after injury. Expression of CXCL12 and CXCR4 in the Ischemic group began to increase on Day 7 and surpassed that in the Normal group after Day 14 (Additional file 1: Figure S1). Western blot analysis confirmed that expression of CXCL12 and CXCR4 was significantly higher in the Normal group than in the Ischemic group on Day 7, at which point infiltration of ASCs was evident (Fig. 2G-I). These data provide evidence that CXCL12 induced early infiltration of ASCs via CXCR4 which mediated the asymmetric regeneration of adipose tissue.

### Migrasomes are detected during asymmetric adipose tissue regeneration

**Fig. 3:**
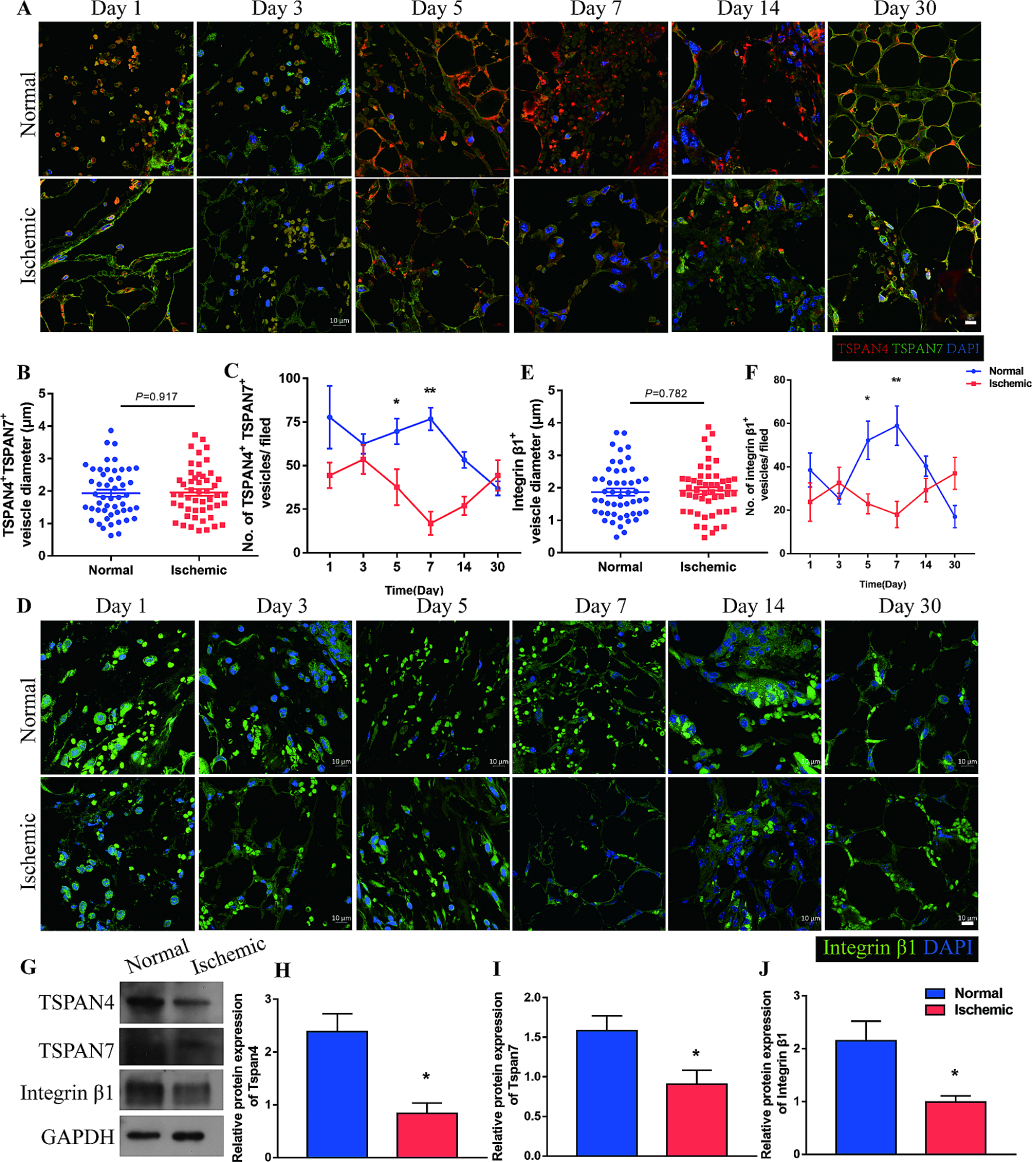
Migrasomes are detected during adipose tissue regeneration. (**A**) Immunofluorescence staining of TSPAN4 and TSPAN7 in adipose tissue from the Normal and Ischemic groups over time. Scale bar, 10 μm. (**B**) Diameters of the TSPAN4^+^ and TSPAN7^+^ vesicles. *n* = 50 vesicles per group. (**C**) Quantitative analysis of TSPAN4^+^ and TSPAN7^+^ vesicles per field in adipose tissue from the Normal and Ischemic group over time points. *p < 0.05, **p < 0.01 compared with Normal. (**D**) Immunofluorescent staining of integrin β1 in adipose tissue from Normal and Ischemic groups over time. Scale bar, 10 μm. (**E**) Diameters of integrin β1^+^ vesicles. *n* = 50 vesicles per group. (**F**) Quantitative analysis of integrin β1^+^ vesicles per field in adipose tissue from the Normal and Ischemic groups over time. *p < 0.05, **p < 0.01 compared with Normal. (**G**) Western blot analysis of TSPAN4, TSPAN7 and integrin β1 protein expression in adipose tissue from Normal and Ischemic groups at 7 days after surgery. (**H-J**) Semiquantitative analysis of TSPAN4, TSPAN7, and integrin β1 protein expression. *p < 0.05 compared with Normal. The data are mean ± SEM. Statistical differences in (**B**), (**E**), (**H**), (**I**) and (**J**) were analyzed using Student’s t test. (**C**) and (**F**) were assessed by One-way ANOVA

Migrating cells aggregate tetraspanin proteins, especially TSPAN4, to form a bulbous membrane on the swelling domains of retraction fibers and finally form migrasomes [29]. Migrasomes can integrate spatial and biochemical information, by which cells are recruited to specific location to exert the function of organ development or tissue regeneration [19–21]. Thus, if migrasomes indeed mediated the establishment of CXCL12 signal in adipose tissue regeneration, the number of migrasomes should correlated with the expression of CXCL12 and recruited ASCs as well as regeneration outcomes between the two sides of adipose repair model. To verify if migrasomes play a role in adipose tissue regeneration, the presence of TSPAN4^+^ and TSPAN7^+^ vesicles was tested in the asymmetric regeneration model. Immunofluorescence staining of tissues detected vesicles enriched with TSPAN4 and TSPAN7 in the extracellular spaces or attached to fibers that projected from cells (Fig. 3A). The average diameter of these vesicles was ∼ 2 μm, which is consistent with the diameter of migrasomes (Fig. 3B). The number of migrasome-like vesicles in the Normal group was higher than that in the Ischemic group as early as Day 1 post-injury, decreased from Day 1 to Day 3, sharply increased until Day 7, and decreased thereafter. However, changes in the number of migrasome-like vesicles were delayed in the Ischemic group. The number of migrasome-like vesicles in the Ischemic group was decreased at Day 5, increased from Day 7, and exceeded that in the Normal group after Day 14 (Fig. 3C).

Enrichment of integrin on the bottom of migrasomes enables their tethering to the extracellular matrix so that they do not move away with cell migration and establish a localized signal along the migrating pathway of cells [30]. To further identify the migrasome-like vesicles, integrin β1 was stained. Vesicles enriched with integrin β1 were observed in the extracellular space, and some of these vesicles were attached to cell projections (Fig. 3D). Integrin β1-enriched vesicles had almost identical diameter distributions as TSPAN4^+^ and TSPAN7^+^ vesicles, and shared a similar exhibition pattern (Fig. 3E, F).

We further tested expression of migrasome markers in tissues. qPCR analysis showed that expression of TSPAN4, TSPAN7, and integrin β1 was elevated in the Normal group during the first 7 days post injury, but began to increase from Day 7 in the Ischemic group and was higher in the Ischemic group than in the Normal group after Day 14 (Additional file 2: Figure S2). Western blot analysis confirmed that higher protein expression of TSPAN4, TSPAN7, and integrin β1 was significantly higher in the Normal group than in the Ischemic group on Day 7 (Fig. 3G-J).

Hence, we detected the presence of migrasomes during adipose tissue regeneration. As expected, the pattern of migrasomes resembled that of CXCL12 expression in adipose tissue after injury.

### ASCs generate migrasomes

**Fig. 4:**
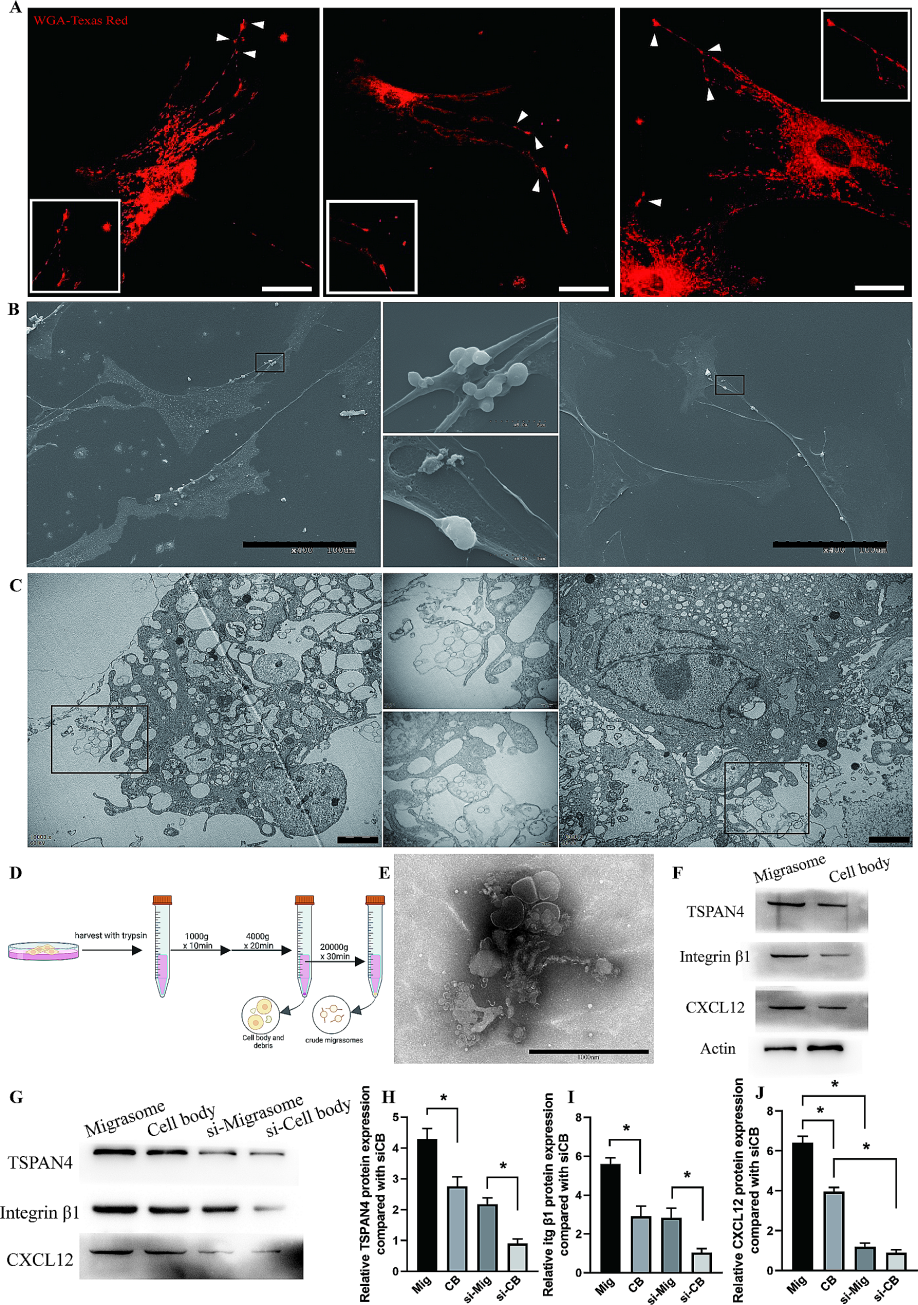
ASCs generate migrasomes. (**A**) Confocal microscopy images of cultured ASCs stained with WGA-Texas Red. Scale bar, 10 μm. White arrows indicated vesicles enriched with WGA that are highly resembled migrasomes, with diameter of ∼ 2 μm and were scattered around ASCs or attached to projections that extended from these cells. (**B**) Scanning electron microscopy images of cultured ASCs. Scale bar, 100 μm. (**C**) TEM images of ASCs cluster. Scale bar, 2 μm. (**D**) Schematic illustration of the centrifugation procedures used to isolate migrasomes. (**E**) TEM image of isolated migrasomes. Scale bar, 1000 nm. (**F**) Western blot analysis of TSPAN4, integrin β1, actin and CXCL12 protein expression in isolated migrasomes. (**G**) Western blot analysis of TSPAN4, integrin β1 and CXCL12 protein expression in isolated migrasomes from ASCs or ASCs treated with siRNA. (**H-J**) Semiquantitative analysis of TSPAN4, Integrin β1, and CXCL12 protein expression. *p < 0.05. The data are mean ± SEM. Statistical differences were analyzed using One-way ANOVA followed by Bonferroni posttest

We noticed that the pattern of migrasomes resembled that of ASCs in the Normal and Ischemic groups (Fig. 2), with infiltration of migrasomes and ASCs delayed in the Ischemic group compared the Normal group. ASCs have been documented to secret CXCL12 under inflammatory conditions [31]. We thus postulated that ASCs generate migrasomes. Fluorescently tagged wheat-germ agglutinin (WGA) is a probe for rapid detection of migrasomes in living cells [32]. We first observed cultured ASCs stained with WGA-Texas Red. Confocal microscopy showed that numerous vesicles enriched with WGA with a diameter of ∼ 2 μm were scattered around ASCs or attached to projections that extended from these cells (Fig. 4A). These vesicles were oval shaped, with diameter that fits the definition of migarsomes and clustered on one side of the cells, proving these vesicles are migrasomes. Live cell time-lapse confocal microscopy also revealed the growth of migrasomes on the tips of or along the retraction fibers generated by ASCs (Additional file 3: Figure S3; Supplementary Video 1). Scanning electron microscopy showed that ASCs generated membrane-bound vesicles with diameters of 0.5–3 μm that were attached to the tips or intersections of retraction fibers, a typical characteristic of migrasomes (Fig. 4B). Transmission electron microscopy (TEM) confirmed that the vesicles were connected or in close proximity to retraction fibers of ASCs. Most of these vesicles were single-membraned, oval, with the diameter of 0.5–3 μm and contained numerous small vesicle (Fig. 4C), which is agreed with the definition of migrasomes. Thus, we showed that ASCs can generate migrasomes.

To confirm the role of ASC-derived migrasomes in adipose tissue regeneration, we first isolated migrasomes from cultured ASCs (Fig. 4D). The isolated migrasomes were analyzed by TEM, which showed a characteristic morphological feature identical to those previously reported, with round shape, attachment to retraction fibers and containing luminal vesicles [19–21, 25, 33] (Fig. 4E). Moreover, the preparations were highly enriched with the migrasome markers TSPAN4 and integrin β1, while expressed lower levels of actin compared with cell body (Fig. 4E). Studies have documented the enrichment of CXCL12 within migrasomes [19–21]. Consistently, migrasomes isolated from ASCs contained significantly higher levels of CXCL12 than the cell bodies (Fig. 4F). In summary, we showed that ASCs can generate migrasomes enriched with CXCL12.

To confirmed the enrichment of CXCL12 within migrasomes and the increased CXCL12 protein expression of migrasomes was indeed mediated by the transferring of CXCL12 from ASCs, we ought to deplete the endogenous CXCL12 expression of ASCs. Using siRNA significantly decreased the protein expression of CXCL12 in ASCs (Additional file 4: Figure S4). Isolated migrasomes from both ASCs and siRNA treated ASCs expressed higher level of migrasome marker TSPAN4 and Integrin β1 compared with cell body (Fig. 4G-I). Migrasomes contained significantly higher level of CXCL12 compared with cell body whereas depleting the expression of CXCL12 within ASCs significantly decreased the CXCL12 level within migrasomes, substantiating the direct relationship between migrasomes and CXCL12 as well as between migrasomes and ASCs (Fig. 4G-J). Furthermore, immunofluorescent stained ASCs with WGA and CXCL12 showed the colocalized enrichment of CXCL12 with WGA, which directly revealed the delivery of CXCL12 by adipose-derived stem cells via migrasomes (Additional file 5: Figure S5).

### Migrasomes promote ASC recruitment and tissue regeneration via CXCR4/RhoA

**Fig. 5:**
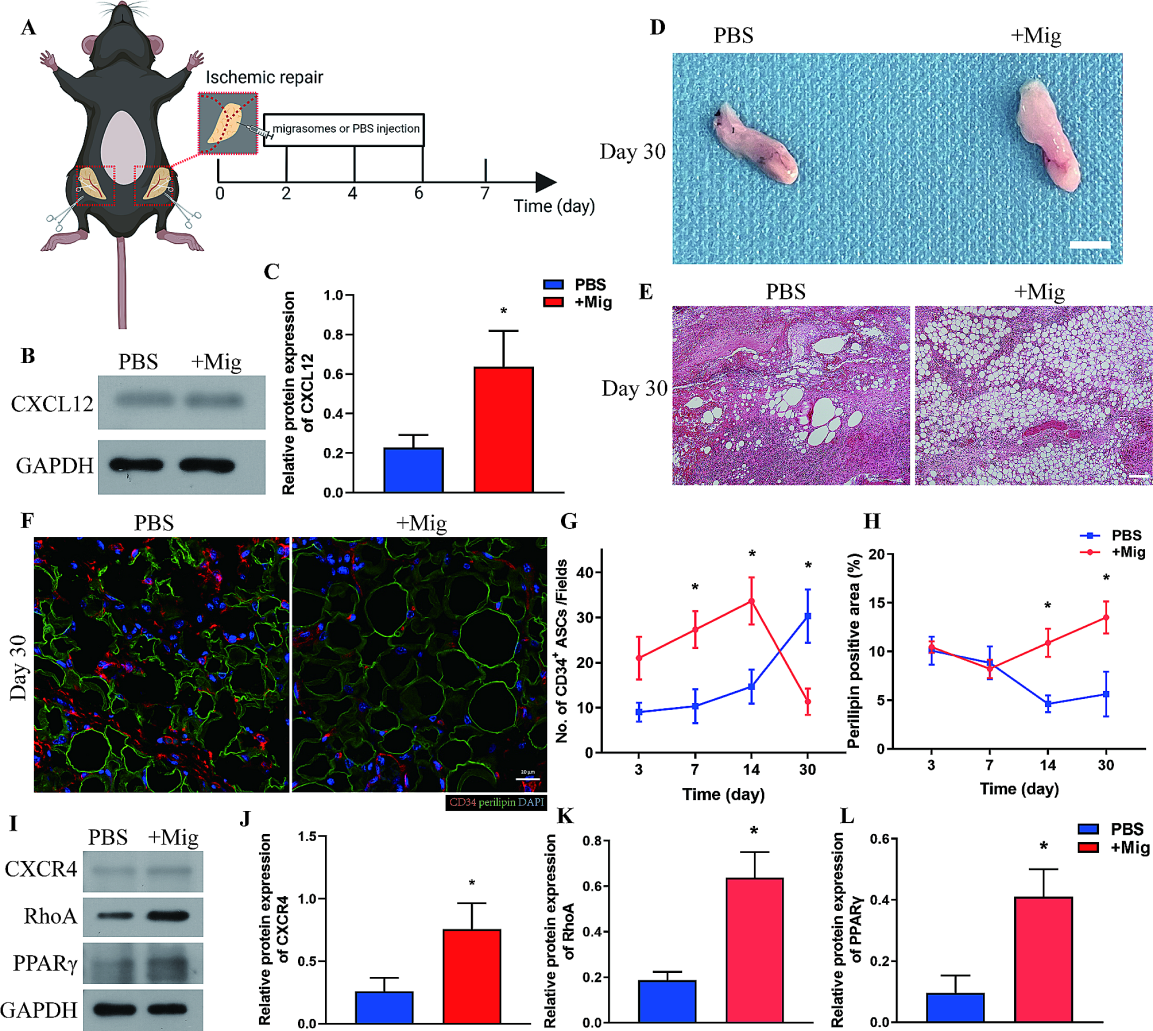
Migrasomes promote adipose tissue regeneration. (**A**) Schematic illustration of injection of poorly vascularized Ischemic adipose tissue with migrasomes (+ Mig group) or PBS (PBS group). *N* = 7 in each groups. (**B**) Western blot analysis of CXCL12 protein expression in adipose tissue from the + Mig and PBS groups at 7 days after surgery. (**C**) Semiquantitative analysis of CXCL12 protein expression. *p < 0.05 compared with PBS group. (**D**) Macroscopic image of adipose tissue from the + Mig and PBS groups at 30 days after surgery. Scale bar, 50 mm. (**E**) HE staining of adipose tissue from the + Mig and PBS groups at 30 days after surgery. Scale bar, 200 μm. (**F**) Immunofluorescence staining of CD34 and perilipin in adipose tissue from the + Mig and PBS groups at 30 days after surgery. Scale bar, 20 μm. (**G**) Quantitative analysis of infiltrated CD34^+^ ASCs per field in adipose tissue from the + Mig and PBS groups over time. (**H**) Quantitative analysis of the perilipin^+^ area per field in adipose tissue from the + Mig and PBS groups over time. *p < 0.05 compared with PBS group. (**I**) Western blot analysis of CXCR4, RhoA, and PPARγ protein expression in adipose tissue from the + Mig and PBS groups 7 days after surgery. (**J-L**) Semiquantitative analysis of CXCR4, RhoA, and PPARγ protein expression. *p < 0.05 compared with PBS group. The data are mean ± SEM. Statistical differences in (**C**) were tested by nonparametric Mann-Whitney test. (**G**), (**H**) and (**J**) to (**L**) were analyzed by One-way ANOVA

Next, we investigated the role of ASC-derived migrasomes in adipose tissue regeneration by adding isolated migrasomes to poorly vascularized adipose tissue post-injury. Migrasomes (+ Mig group) or phosphate-buffered saline (PBS, PBS group) were focally injected into inguinal fat pads (Ischemic repair model) subjected to the same procedure as the Ischemic group for six consecutive days post-injury (Fig. 5A). Migrasomes, including stem cell-derived migrasomes, are enriched with CXCL12 [19, 21, 22]. Consistently, western blot analysis showed that expression of CXCL12 was significantly higher in the + Mig group than in the PBS group at Day 7 after injury (Fig. 5B, C). Macroscopic observation on Day 30 showed that tissues in the + Mig group had a more natural appearance and soft texture while those in the PBS group appeared atrophic and felt rigid (Fig. 5D). HE staining of adipose tissues on Day 30 indicated that adipose tissue regeneration was better in the + Mig group than in the PBS group. Large vacuoles, oil cyst-like structure, and severe fibrosis were observed in the PBS group, whereas regularly arranged round adipocytes of different sizes were observed in the + Mig group (Fig. 5E).

To verify the mechanism by which migrasomes promote tissue regeneration, immunofluorescence staining of CD34 and perilipin was performed. Quantification of CD34^+^ cells showed that migrasomes promote the infiltration of ASCs as early as Day 3 post-injury. The number of ASCs sharply increased from Day 3 to Day 14 in the + Mig group but remained relatively low in the PBS group. The number of ASCs decreased in the + Mig group but increased in the PBS group from Day 14 to Day 30, indicating ASCs are required for regenerative events in PBS group (Fig. 5F, G). The perilipin^+^ area remained low in both groups in the first 7 days. The perilipin^+^ area in the + Mig group increased from Day 7 to Day 30. By contrast, the perilipin^+^ area in the PBS group dropped from Day 7 to Day 14 and slightly increased to Day 30 (Fig. 5F, H). Tissue structures remained broken with significantly more infiltrated ASCs in the PBS group, while more complete tissue structures with fewer ASCs were observed in the + Mig group (Fig. 5F). In summary, we showed that migrasomes can promote adipose tissue regeneration by facilitating early recruitment of ASCs.

CXCL12 binds to its receptor CXCR4 and activates RhoA through activation of the small G proteins, Gi and Gα13, which leads to directional cell migration [34, 35]. Western blot analysis of tissues on Day 7 confirmed that protein expression of CXCR4 and RhoA was significantly higher in the + Mig group than in the PBS group (Fig. 5I-K), indicating that migrasomes activate CXCR4/RhoA signaling. Furthermore, addition of migrasomes promoted expression of the adipogenesis-associated protein PPAR-γ (Fig. 5I, L). These data show that migrasomes enriched with CXCL12 promote adipose tissue regeneration by recruiting ASCs through CXCR4/RhoA signaling. The recruited ASCs augment tissue regeneration probably by promoting adipogenesis as shown by elevated expression of PPAR-γ.

### Blockade of ASC infiltration reduces the number of migrasomes

**Fig. 6:**
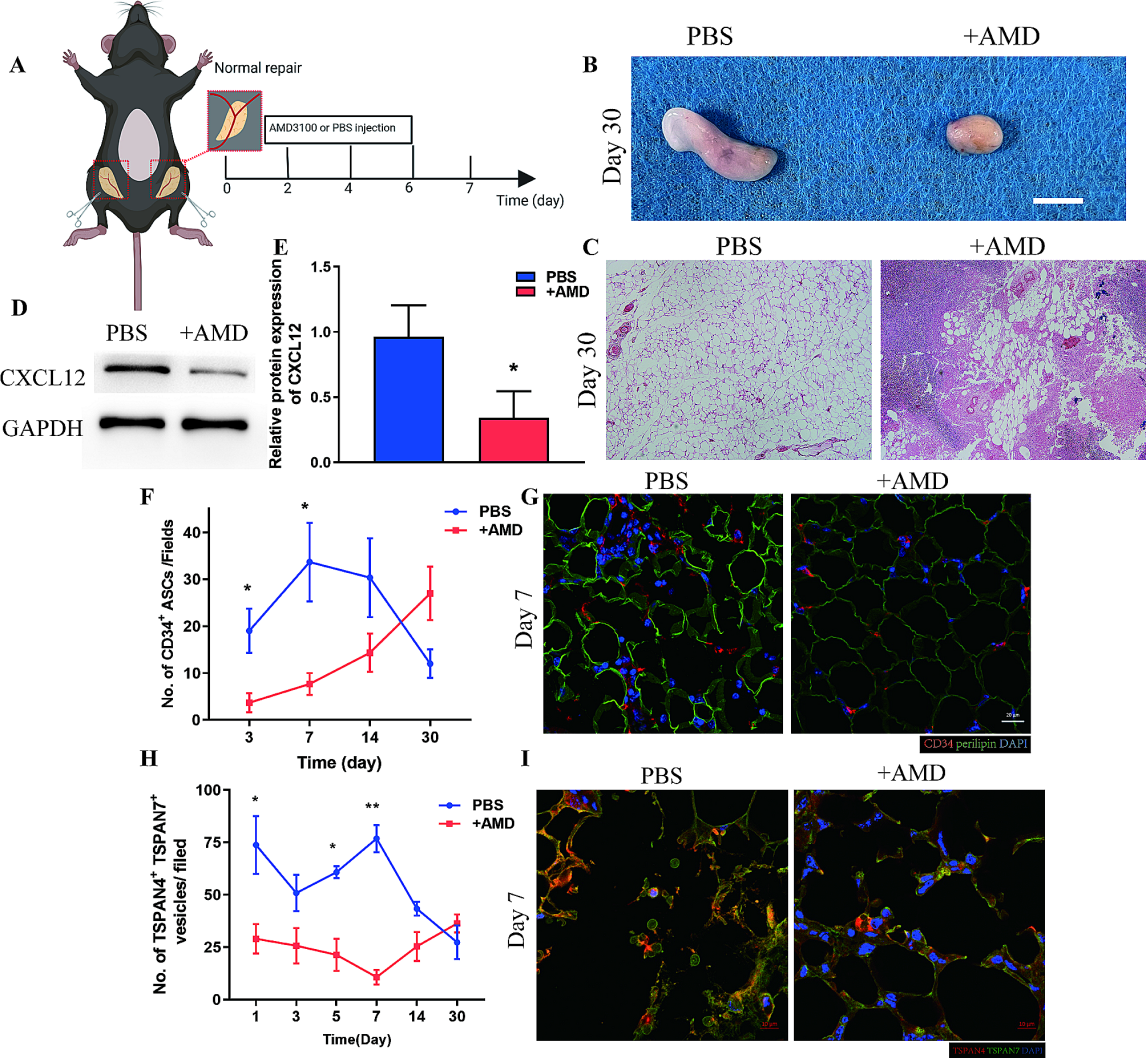
ASCs generate migrasomes in vivo. (**A**) Schematic illustration of inhibition of ASC infiltration in Normal adipose tissue using CXCR4 inhibitor AMD3100 (+ AMD). *N* = 7 in each groups. (**B**) Macroscopic image of adipose tissue from the PBS and + AMD groups at 30 days after surgery. Scale bar, 50 mm. (**C**) HE staining of adipose tissue from the PBS and + AMD groups at 30 days after surgery. Scale bar, 200 μm. (**D**) Western blot analysis of CXCL12 protein expression in adipose tissue from the PBS and + AMD groups at 7 days after surgery. (**E**) Semiquantitative analysis of CXCL12 protein expression. *p < 0.05 compared with PBS group. (**F**) Immunofluorescence staining of CD34 and perilipin in adipose tissue from the + AMD and PBS groups at 7 days after surgery. Scale bar, 20 μm. (**G**) Quantitative analysis of infiltrated CD34^+^ ASCs per field in adipose tissue from the + AMD and PBS groups over time. *p < 0.05 compared with PBS. (**H**) Immunofluorescence staining of TSPAN4 and TSPAN7 in adipose tissue from the + AMD and PBS groups at 7 days after surgery. Scale bar, 10 μm. (**I**) Quantitative analysis of migrasomes per field in adipose tissue from the + AMD and PBS groups over time. *p < 0.05, **p < 0.01 compared with PBS. The data are mean ± SEM. Statistical differences were analyzed using One-way ANOVA

To prove the origin of migrasomes in vivo, we blocked infiltration of ASCs using the CXCR4 inhibitor AMD3100 [24]. AMD3100 (+ AMD group) or PBS (PBS group) was focally injected to the inguinal fat pads subjected to the same procedure as the Normal group (Fig. 6A). The in vivo use of AMD3100 prevent the infiltration of ASCs, which worsen the regeneration of adipose tissue. Macroscopic observation on Day 30 post operation showed adipose tissue from + AMD group with decreased tissue mass and atrophy appearance and brittle texture while that from PBS group with natural appearance and soft texture (Fig. 6B). HE staining showed tissue structure on Day 30 from PBS group was stable, with round adipocytes arranged tightly and regularly whereas large vacuoles, severe cell infiltration with disorganized structure could be witnessed from adipose tissue of + AMD group (Fig. 6C). Furthermore, western blot analysis of tissue from Day 7 showed expression of CXCL12 was significantly higher in the PBS group than in the + AMD group (Fig. 6D-E). Quantitative analysis of CD34^+^ cells confirmed that AMD3100 blocked infiltration of ASCs, with significantly lower levels of CD34^+^ cells in the + AMD group than in the PBS group over the first 7 days (Fig. 6B, C). Immunofluorescence staining of TSPAN4 and TSPAN7 confirmed that blockade of ASC infiltrations significantly decreased the number of migrasomes during tissue regeneration (Fig. 6D, E). These data provide evidence that ASCs can generate migrasomes enriched with CXCL12 to promote ASC infiltration during adipose tissue regeneration.

### Migrasomes enhance migration of ASCs via CXCR4/RhoA in vitro

**Fig. 7:**
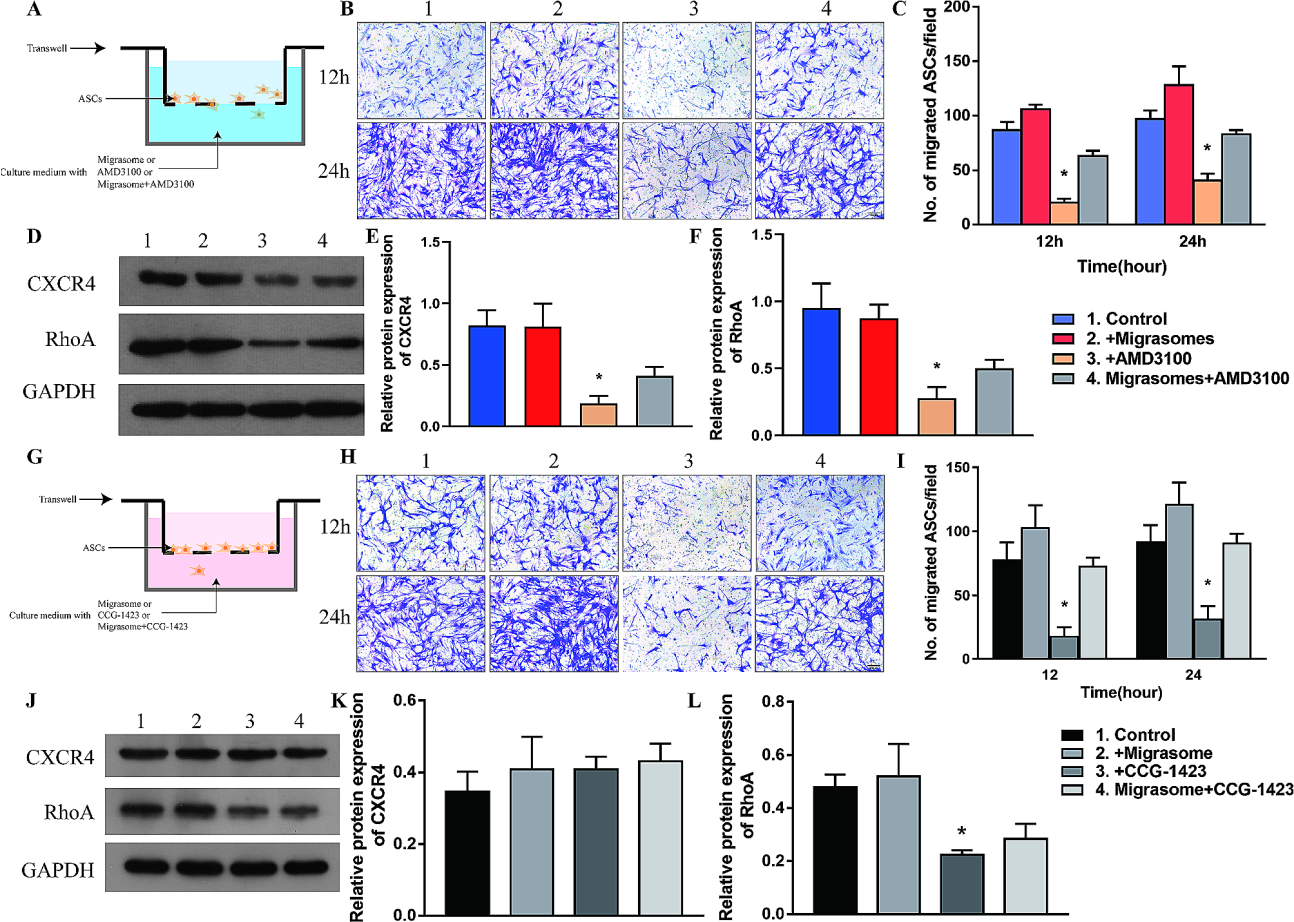
Migrasomes promote migration of ASCs in vitro via activation of CXCR4/RhoA signaling by CXCL12. (**A**) Schematic illustration of the transwell assay of ASCs with the control, migrasomes, AMD3100, and Migrasome + AMD3100 groups. Data are obtained from 3 independent reduplicated experiments. (**B**) Images of migrated ASCs after incubation for 12 and 24 h. Scale bar, 200 μm. (**C**) Quantitative analysis of the number of migrated ASCs per field. *p < 0.05 compared with control. (**D**) Western blot analysis of CXCR4 and RhoA protein expression. (**E-F**) Semiquantitative analysis of CXCR4 and RhoA protein expression. *p < 0.05 compared with control. (**G**) Schematic illustration of the transwell assay of ASCs with the control, migrasomes, CCG-1423 or Migrasomes + CCG-1423 groups. Data are obtained from 3 independent reduplicated experiments. (**H**) Images of migrated ASCs after incubation for 12 and 24 h. Scale bar, 200 μm. (**I**) Quantitative analysis of the number of migrated ASCs per field. *p < 0.05 compared with control. (**J**) Western blot analysis of CXCR4 and RhoA protein expression. (**K-L**) Semiquantitative analysis of of CXCR4 and RhoA protein expression. *p < 0.05 compared with control. The data are mean ± SEM. Statistical differences in (**C**) was assessed by Kruskal-Wallis. (**E**), (**F**), (**I**), (**K**) and (**L**) were analyzed using One-way ANOVA followed by Bonferroni posttest

To verify the capacity of migrasomes to promote migration of ASCs, we performed transwell assay with migrasomes, AMD3100, and migrasomes plus AMD3100 (migrasome + AMD3100 group) (Fig. 7A). Migration capacity of ASCs was enhanced by migrasomes, diminished by AMD3100, and restored in the migrasome + AMD3100 group (Fig. 7B, C). Western blot and qPCR analyses of cultured cells showed that expression of CXCR4 and RhoA was upregulated by migrasomes and inhibited by AMD3100 (Fig. 7D-F, Additional file 6: Figure S6). Expression of CXCR4 and RhoA was partially restored in the migrasome + AMD3100 group, suggesting that migrasomes promote migration of ASCs via CXCR4/RhoA signaling.

The transwell assay was performed with control, migrasomes, CCG-1423 (a RhoA inhibitor), and migrasomes plus CCG-1423 (migrasome + CCG-1423 group) (Fig. 7G). Migration of ASCs was promoted by migrasomes, diminished by CCG-1423, and restored in the migrasome + CCG-1423 group (Fig. 7H, I). Western blot and qPCR analyses showed that migrasomes increased expression of RhoA, while CCG-1423 inhibited expression of RhoA and had no influence on expression of CXCR4 (Fig. 7J-L, Additional file 7: Figure S7). Expression of RhoA was partially rescued while expression of CXCR4 was not significantly altered in the migrasome + CCG-1423 group (Fig. 7J-L, Figure S7). In summary, we demonstrated that migrasomes enriched with CXCL12 promote migration of ASCs in vitro via CXCR4/RhoA signaling.

## Discussion

As newly discovered membrane-bound vesicles, migrasomes have been reported to promote recruitment of CXCR4^+^ cells during tissue development by carrying the chemokine CXCL12 [19–21]. To the best of our knowledge, no reports on whether migrasomes participate in soft tissue regeneration has been reported yet. In this study, we showed for the first time that ASCs could generate migrasomes, which play a key role in adipose tissue regeneration. Migrasomes derived from ASCs were enriched with CXCL12 to promote recruitment of ASCs and adipose tissue regeneration. Migrasomes promoted migration of ASCs through CXCL12-activated CXCR4/RhoA signaling in vivo and in vitro, an effect that could be abrogated by CXCR4 inhibitor AMD3100. Treatment of poor vascularized adipose tissue with ASC-derived migrasomes augmented stem cell recruitment and enhanced tissue regeneration, indicating that ASC-derived migrasomes are a new tool to restore soft tissue regeneration.

CXCL12 is a highly important chemokine for the viability and mobility of stem cells on its interaction with CXCR4 which have been proved expressing on ASCs [36, 37]. Kato et al. demonstrated the suppressed expression of CXCL12 contributed to the impaired migration of ASCs in glucocorticoid induced delayed wound healing and CXCL12 overexpression restored the wound healing potential of ASCs [38]. Furthermore, Fadera et al. showed the higher expression of CXCR4 was consistent with the promotive migration of ASCs to CXCL12 and could be an ideal strategy for ischemic diseases treating [39]. Thus, understanding the mechanism of the elevated level of CXCL12 represents a promising target for regulating ASCs infiltration after adipose tissue damage. The fact that many studies have shown migrasomes could package CXCL12 and the delicate pattern of CXCL12 delivering by migrasomes during morphogenesis in zebrafish gastrulation [19] led us to explore whether migrasomes deposit CXCL12 during adipose tissue regeneration. The unique labeling and characteristics of migrasomes in vivo is to detect the TSPAN4 as well as integrin β1 positive vesicles that are connected to projections from cells [19]. Consistently, we found numerous of TSPAN4 positive vesicles that were connected to the tubular structures extended from cells among the extracellular spaces during adipose tissue regeneration. Quantification analysis of the diameters of these vesicles agreed with the characteristics of migrasomes, with diameters from 0.5 to 3 μm. As the key genes mediate migrasomes formation, the expression level of TSPAN4, TSPAN7 and Integrin β1 also corelated to the results of tissue immunofluorescent staining, which further confirmed that migrasomes participate adipose regeneration.

Here, we showed that the pattern of migrasomes during adipose tissue regeneration highly coincided with those of CXCL12 expression and ASC infiltration. We thus postulated that ASCs generated migrasomes containing CXCL12 during adipose regeneration. Indeed, the migration of ASCs to damaged tissue has been illustrated under the conditions of muscle regeneration, cancer progression and adipose regeneration, suggesting the potential of generating migrasomes by ASCs in vivo [40–42]. The in vitro characterization assays confirmed that ASCs could generate migrasomes. Scanning electron microscopy showed ASCs generate membrane structures with oval shape and diameters from 0.5 to 3 μm that tend to cluster on one side of the cell and are connected or closely related to the retraction fibers. TEM analysis showed these vesicles are large vesicles that contain smaller vesicles within their lumen, with one membrane that connected to the projections of migrating cells. These characteristics distinguished a lot from MVBs, which fuse with the plasma membrane to release exosomes and exosomes, which are cell-derived small vesicles present in biological fluids with diameters less than 150 nm [20, 43, 44]. The role of ASCs derived migrasomes was further verified in vivo. Assisting ischemic adipose tissue regeneration with ASCs derived migrasomes rescued the early and higher expression of CXCL12, which recruit more ASCs and enhance tissue regeneration. Using AMD3100 inhibited the infiltration of ASCs and migrasomes, which prevent the upregulation of CXC12 post adipose tissue regeneration even when the vessel was intact. Unlike exosomes that are released to the biological fluids. Migrasomes carrying chemical signals can be deposited along the pathway of migrating cells and anchored via abundant integrin expression, enabling the establishment of a diffusion-restricted, prolonged signal pattern that integrate biological and spatial signals [19–21]. Release of a signal from migrasomes can elicit a latent effect because it requires membrane rupture or leakage or taken up by recipient cells, which might further fine-tune the signal pattern [21]. Thus, deposition of migrasomes during adipose tissue regeneration might be the mechanism responsible for the precise migration of ASCs both spatially and temporally.

Cell migration requires actin cytoskeletal reorganization, which is mediated by Rho signaling. RhoA stimulates myosin-based retraction of the cell rear and generates the force needed for retraction of the trailing edge during migration [34]. The CXCL12/CXCR4 chemokine signaling activated RhoA has been a well-documented driver for cell migration. Binding to CXCL12 induced a conformational change of the G protein coupled receptor CXCR4 and dissociation of Gα from Gβ subunits. The activated Gα13 could stimulate PDZ-RhoGEF and subsequently activate RhoA [35]. CXCL12/CXCR4 has also been shown upregulate LncRNA XIST, which functioned as ceRNA sponging miR-133a-3p to promote RhoA expression [45]. RhoA is also documented as the core modulator of CXCL12/CXCR4-mediated migration of stem cell, including ASCs [35, 46, 47]. Consistently, our study demonstrated that migrasomes promote migration of ASCs via activation RhoA signaling by CXCL12 through CXCR4, as evidenced by promotion of ASCs migration in transwell assay and upregulation of RhoA expression, a process that could be inhibited by a RhoA inhibitor. Upregulation of CXCR4 and RhoA as well as ASC recruitment upon addition of migrasomes to poorly regenerated tissue confirmed CXCR4/RhoA are part of the mechanism by which migrasomes attract ASCs in vivo.

ASCs have been widely applied and are indispensable in any types of adipose tissue reconstruction. The number of ASCs parallels the outcomes of adipose tissue regeneration, and the therapeutic effects of ASCs are largely attributed to their paracrine effect, which counteracts degeneration post-injury [8]. ASCs could paracrine proangiogenic factors such as HGF, VEGF, the expression of which were upregulated under hypoxia [48]. Furthermore, the beneficial effect of ASCs on angiogenesis could also be strengthened by CXCL12. In vitro study did show ASCs acquire EC marker and form capillary structures via a CXCL12/CXCR4 dependent pathway [49]. Hypoxia promote the reaction of ASCs to CXCL12 by upregulating the expression of CXCR4, which might further recruit more ASCs for angiogenesis [48]. Besides, CXCL12 has been shown to be capable of mobilizing and recruiting CXCR4 positive endothelia progenitor cells (EPCs) for the required angiogenesis and vasculogenesis, and the therapeutic application of CXCL12 for ischemic related disorders has been well documented [50, 51, 52]. Thus, CXCL12 might facilitate angiogenesis during adipose tissue regeneration by recruiting EPCs as well as ASCs and exert synergistic effect on the therapeutic functions of ASCs. Herein, we have showed that ASCs derived migrasomes were rich with CXCL12. Assisting tissue repair with ASCs derived migrasomes (+ Mig) lead to the early higher expression of CXCL12 and recruitment of ASCs post adipose tissue damage. To be noticed, the percentage of perilipin positive area within adipose tissue surged at Day 7 in + Mig group, suggesting the better angiogenesis that mitigate ischemia condition within tissue, since adipocyte could not survive ischemia [27]. It is controversial whether ASCs promote adipogenesis by directly differentiate into mature adipocytes [53, 54]. Nevertheless, ASCs elevated expression of the adipogenesis-associated protein PPARγ, which is a key regulator of adipogenesis that determines the commitment of preadipocytes to become mature adipocytes [55]. Our work further highlights the importance of ASCs in adipose tissue regeneration as they create a positive feedback loop by depositing migrasomes enriched with CXC12, which might sustain the required spatial and temporal recruitment of ASCs during regeneration.

**Fig. 8:**
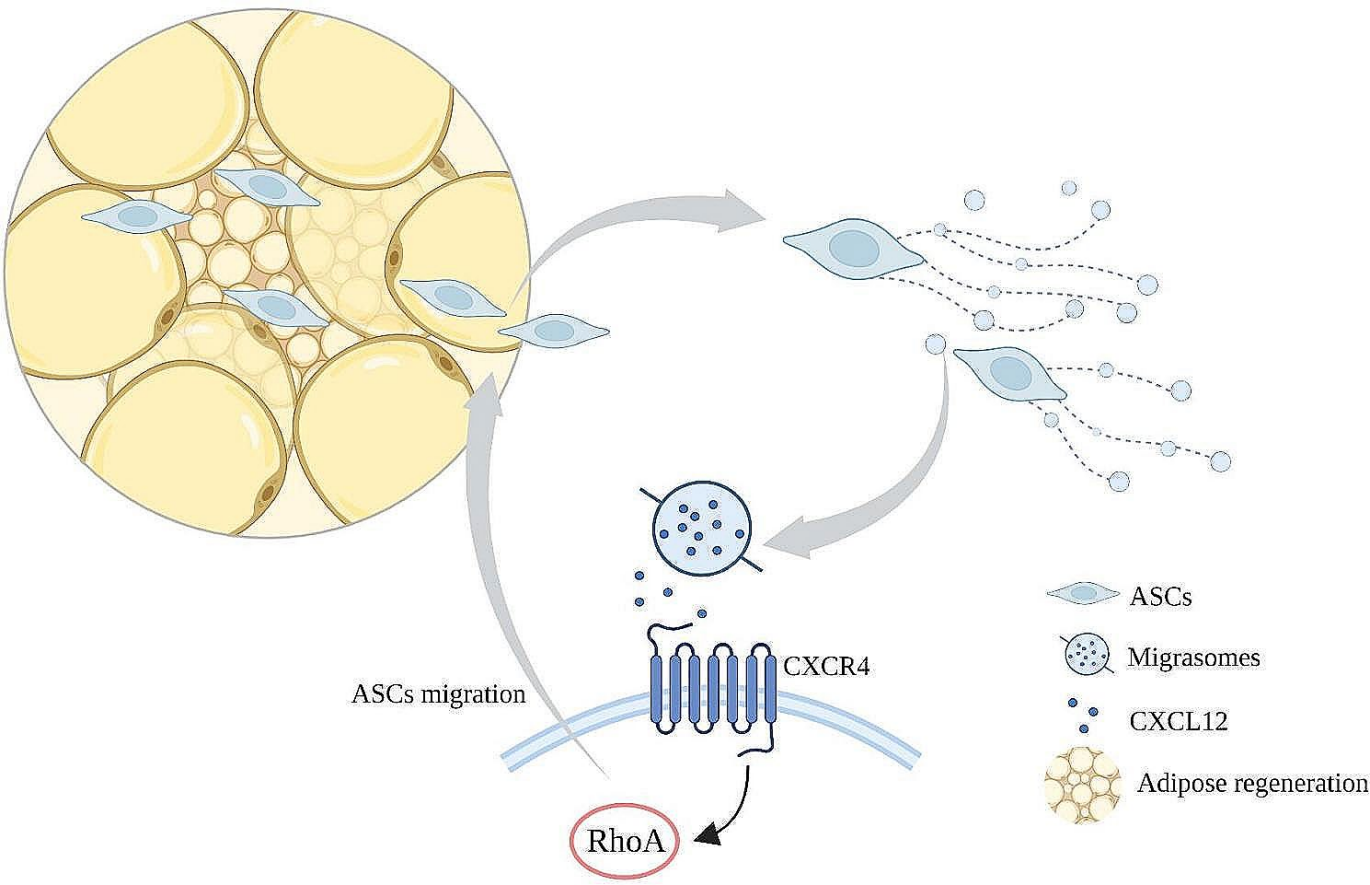
Graphic illustration of ASC-derived migrasomes mediating soft tissue regeneration

In summary, we demonstrated that ASCs generate migrasomes enriched with CXCL12 and plays a pivotal role in activating CXCR4/RhoA signaling to recruit ASCs during adipose tissue regeneration (Fig. 8). Importantly, ASC-derived migrasomes can restore regeneration of poorly vascularized tissue, and may be a new therapeutic target for soft tissue regeneration. In addition to adipose tissue regeneration, ASCs have been applied to facilitate the treatment of different degenerative settings such as diabetic wound healing, musculoskeletal regeneration, and neurodegenerative diseases [56]. Many of their therapeutic functions rely on secretion of cytokines, growth factors and chemokines. Migrasomes might also play an important role in these processes, but remains to be thoroughly elucidated. Furthermore, recent study has demonstrated that mesenchymal stem cell-derived migrasomes carry the cell surface molecules of the parent cells [22]. Cultured ASCs were generally not immunogenic, which result from the decline of antigen presenting cells (APCs) within cultured ASCs and the absence of APC-related surface proteins on ASCs with the progressive passage [57]. This might further endowed ASCs-derived migrasomes with the advantages as tools to be used allogeneic in regenerative medicine, which could be the focus of further research.

## Conclusions

This work uncovers a previously unknown cell function of ASCs in tissue regeneration through generating migrasomes. Migrasomes from ASCs are rich in chemokine CXCL12, which in turn chemoattract stem cells by activating CXCR4/RhoA signal and augment adipose tissue regeneration. These finding indicate ASC-derived migrasomes as a new therapeutic target in ASC-mediated tissue regeneration and may obtain boarder applications in the regenerative medicine.

### Electronic supplementary material

Below is the link to the electronic supplementary material.

Supplementary Material 1

Supplementary Material 2

Supplementary Material 3

Supplementary Material 4

Supplementary Material 5

Supplementary Material 6

Supplementary Material 7

Supplementary Material 8

Supplementary Material 9

## Data Availability

No datasets were generated or analysed during the current study.

## Abbreviations

ASCs: Adipose derived stem cells
CXCL12: Chemokine (C-X-C motif) ligand 12
CXCR4: Chemokine C-X-C-Motif Receptor 4
TSPAN4: Tetraspanin 4
TSPAN7: Tetraspanin 7
PPARγ: Peroxisome proliferator-activated receptorγ
RhoA: Ras homolog gene family member A
DAPI: 4’,6-diamidino-2-phenylindole
GAPDH: Glyceraldehyde-3-phosphatedehydrogenase
TEM: Transmission electron microscopy
HE: Hematoxylin and eosin
PBS: phosphate-buffered saline
APC: antigen presenting cell
