# Supplementary material for: Migrasomes from adipose derived stem cells enrich CXCL12 to recruit stem cells via CXCR4/RhoA for a positive feedback loop mediating soft tissue regeneration
Source: J Nanobiotechnology. 2024 May 3;22:219. doi: 10.1186/s12951-024-02482-9 (PMC11067256; doi:10.1186/s12951-024-02482-9)
Supplement: Supplementary file 1 — Supplementary Material 1 [file 12951_2024_2482_MOESM1_ESM.pdf]

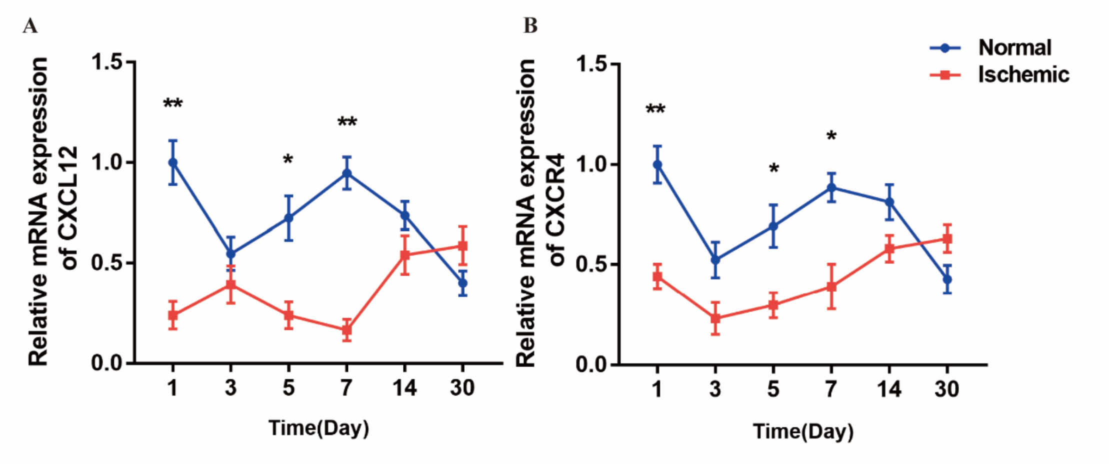

Figure S1. Relative mRNA expression of CXCL12 and CXCR4 in adipose tissue from the Normal and Ischemic groups over time. (A) mRNA expression of CXCL12 over time. **(B)** mRNA expression of CXCR4 over time. $*p < 0.05$, $**p < 0.01$ compared with Normal. The data are mean ± SEM. Statistical differences were assessed using One-way ANOVA.
