# Supplementary material for: Migrasomes from adipose derived stem cells enrich CXCL12 to recruit stem cells via CXCR4/RhoA for a positive feedback loop mediating soft tissue regeneration
Source: J Nanobiotechnology. 2024 May 3;22:219. doi: 10.1186/s12951-024-02482-9 (PMC11067256; doi:10.1186/s12951-024-02482-9)
Supplement: Supplementary file 2 — Supplementary Material 2 [file 12951_2024_2482_MOESM2_ESM.pdf]

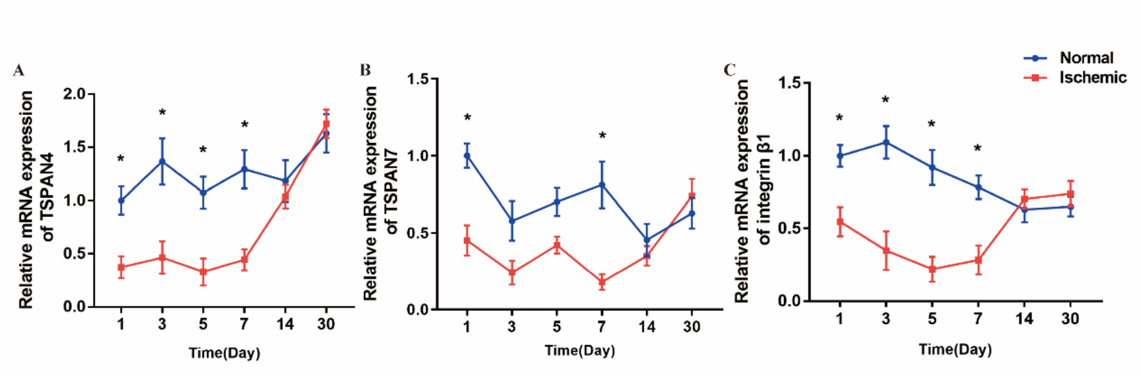

Figure S2. Relative mRNA expression of TSPAN4, TSPAN7, and integrin β1 in adipose tissue from the Normal and Ischemic groups over time. (A) Relative mRNA expression of TSPAN4 over time. **(B)** Relative mRNA expression of TSPAN7 over time. **(C)** Relative mRNA expression of integrin β1 over time. *$p < 0.05$, **$p < 0.01$ compared with Normal. The data are mean ± SEM. Statistical differences were analyzed using One-way ANOVA.
