# Supplementary material for: Migrasomes from adipose derived stem cells enrich CXCL12 to recruit stem cells via CXCR4/RhoA for a positive feedback loop mediating soft tissue regeneration
Source: J Nanobiotechnology. 2024 May 3;22:219. doi: 10.1186/s12951-024-02482-9 (PMC11067256; doi:10.1186/s12951-024-02482-9)
Supplement: Supplementary file 3 — Supplementary Material 3 [file 12951_2024_2482_MOESM3_ESM.pdf]

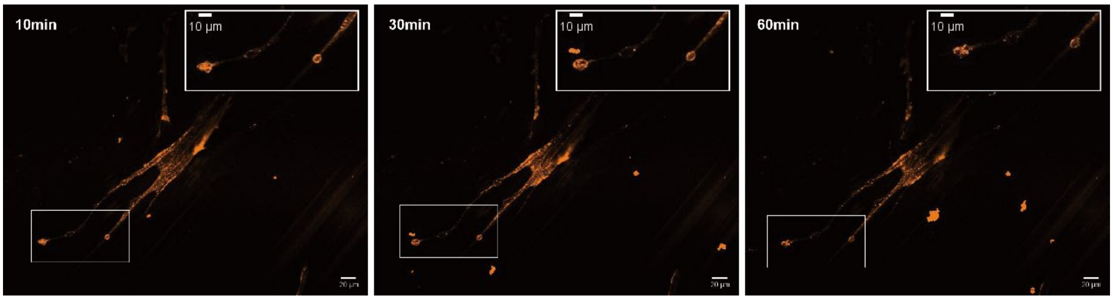

Figure S3. Live cell time-lapse confocal microscopy of ASCs. Cultured ASCs were stained with WGA and then observed by time-lapse confocal microscope. Magnified images of the growth of migrasomes on the tips of the retraction fibers generated by ASCs is shown in the attached boxes.
