# Supplementary material for: Migrasomes from adipose derived stem cells enrich CXCL12 to recruit stem cells via CXCR4/RhoA for a positive feedback loop mediating soft tissue regeneration
Source: J Nanobiotechnology. 2024 May 3;22:219. doi: 10.1186/s12951-024-02482-9 (PMC11067256; doi:10.1186/s12951-024-02482-9)
Supplement: Supplementary file 4 — Supplementary Material 4 [file 12951_2024_2482_MOESM4_ESM.pdf]

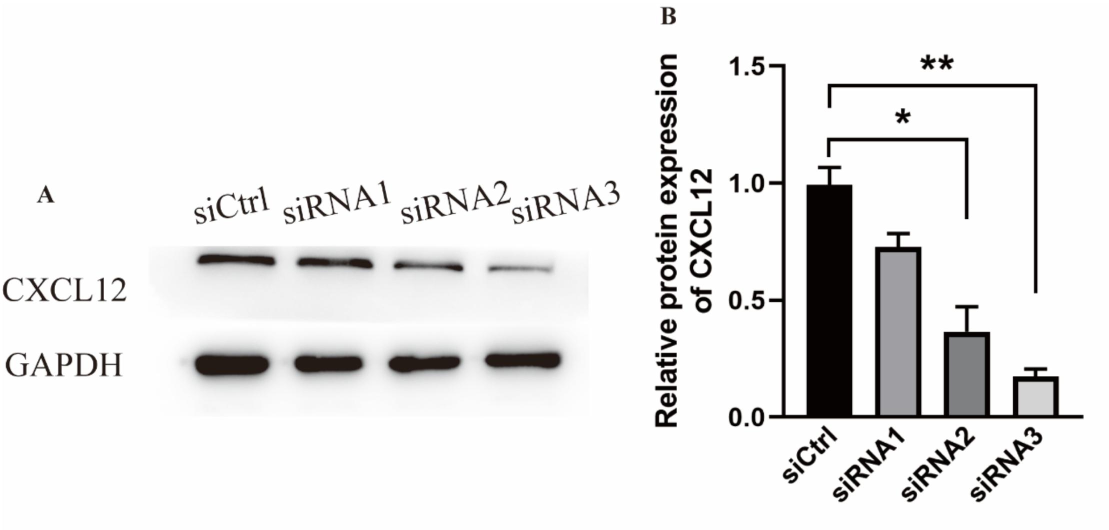

Figure S4. Western blot analysis of CXCL12 protein expression in ASCs treated with siRNA. **(A)** Western blot analysis of CXCL12 in ASCs treated with siRNA. **(B)** Relative protein expression of CXCL12 in ASCs treated with siRNA. The data are mean ± SEM. *p < 0.05. Statistical differences were analyzed using One-way ANOVA followed by Bonferroni posttest.
