# Supplementary figures and images for: Migrasomes from adipose derived stem cells enrich CXCL12 to recruit stem cells via CXCR4/RhoA for a positive feedback loop mediating soft tissue regeneration
Source: J Nanobiotechnology. 2024 May 3;22:219. doi: 10.1186/s12951-024-02482-9 (PMC11067256; doi:10.1186/s12951-024-02482-9)

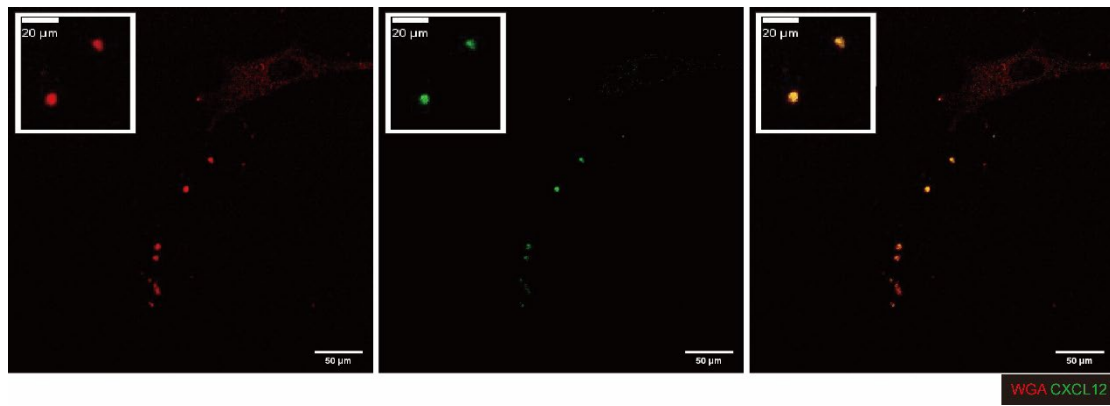

**Figure S5. Immunofluorescent stained ASCs with WGA and CXCL12.**

Supplement: Supplementary file 5 — Supplementary Material 5 [file 12951_2024_2482_MOESM5_ESM.pdf]
