# Supplementary material for: Migrasomes from adipose derived stem cells enrich CXCL12 to recruit stem cells via CXCR4/RhoA for a positive feedback loop mediating soft tissue regeneration
Source: J Nanobiotechnology. 2024 May 3;22:219. doi: 10.1186/s12951-024-02482-9 (PMC11067256; doi:10.1186/s12951-024-02482-9)
Supplement: Supplementary file 7 — Supplementary Material 7 [file 12951_2024_2482_MOESM7_ESM.pdf]

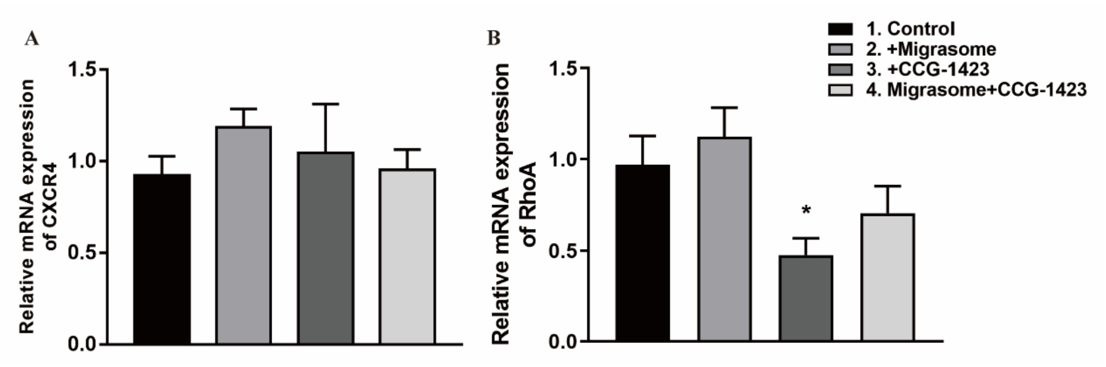

Figure S7. Relative mRNA expression of CXCR4 and RhoA in ASCs cultured in the control, migrasomes, CCG-1423 or Migrasome+CCG-1423 groups. (A) Relative mRNA expression of CXCR4. **(B)** Relative mRNA expression of RhoA. The data are mean ± SEM. *$p < 0.05$. Statistical differences were analyzed using One-way ANOVA followed by Bonferroni posttest.
