# Supplementary material for: Migrasomes from adipose derived stem cells enrich CXCL12 to recruit stem cells via CXCR4/RhoA for a positive feedback loop mediating soft tissue regeneration
Source: J Nanobiotechnology. 2024 May 3;22:219. doi: 10.1186/s12951-024-02482-9 (PMC11067256; doi:10.1186/s12951-024-02482-9)
Supplement: Supplementary file 9 — Supplementary Material 9 [file 12951_2024_2482_MOESM9_ESM.docx]

**Table supplementary 1**

| TSPAN4-F1 | GACAGTTATGCCCAACAAGAC |
| --- | --- |
| TSPAN4-R1 | ACACGAGTGGCATTGTATACC |
| TSPAN7-F1 | CTGCATGAACGAAACTGACT |
| TSPAN7-R1 | AGCAATGATCCCCATGTTAG |
| integrinβ1-F1 | GATCCTGTGACCCATTGCA |
| integrinβ1-R1 | AACCACGCCTGCTACAATT |
| CXCR4-F1 | GAGAGCATCGTGCACAAGTG |
| CXCR4-R1 | CCTCTGCTCATGGAGTTGAG |
| CXCL12-F1 | GTGGGTTCTGTTTATCCTCTCA |
| CXCL12-R1 | GGTAGCTGTTTGGAGAGAATG |
| RhoA-F1 | AGCCTGTGGAAGACATGCTT |
| RhoA-R1 | TCAAACACTGTGGGCACATAC |
| GAPDH-F1 | GGCCTCCAAGGAGTAAGAAA |
| GAPDH-R1 | GCCCCTCCTGTTATTATGG |
